# Organizational Silence as a Mediator Between Ethical Nursing Competence and Perceptions of Open Disclosure in Patient Safety Incidents

**DOI:** 10.1155/jonm/3804799

**Published:** 2026-03-29

**Authors:** Yuna Kim, Youn-Jung Son, Sun Joo Jang, Haeyoung Lee

**Affiliations:** ^1^ Graduate School of Nursing and Health Professions, Chung-Ang University, Seoul, Republic of Korea, cau.ac.kr; ^2^ Red Cross College of Nursing, Chung-Ang University, Seoul, Republic of Korea, cau.ac.kr; ^3^ College of Nursing and the Research Institute of Nursing Science, Seoul National University, Seoul, Republic of Korea, snu.ac.kr

**Keywords:** ethical competence, nurses, open disclosure, organizational silence, patient safety incidents

## Abstract

**Background:**

Open disclosure of patient safety incidents (PSIs) is essential for patient safety, but organizational silence may inhibit nurses from speaking up. However, the influence of ethical competence on the perception of open disclosure and the role of organizational silence remain unclear. This study aimed to examine the relationships among nurses’ ethical competence, organizational silence, and perceptions of open disclosure in PSIs and whether organizational silence mediates the effect of ethical competence on perceptions of open disclosure.

**Methods:**

A cross‐sectional survey of 303 nurses in South Korea measured their ethical competence, organizational silence, and perceptions of open disclosure. Data were collected between October 14 and November 8, 2024, via an online nursing community. Multiple regression and mediation analyses were used to test for direct and indirect effects while adjusting for covariates.

**Results:**

Ethical competence was significantly associated with lower organizational silence and higher open disclosure perception, whereas organizational silence was associated with lower open disclosure perception. Mediation analysis confirmed that organizational silence had a significant indirect effect.

**Conclusions:**

Higher ethical competence is linked to lower organizational silence and greater support for the perception of open disclosure. Enhancing nurses’ ethical competence and reducing silence may foster a more transparent patient safety culture.

**Implications for nursing management:**

Nurse managers should strengthen nurses’ ethical competence, encourage open communication to reduce silence, and promote transparent disclosure of PSIs.

## 1. Introduction

The global healthcare environment is inherently complex and uncertain, thereby fostering a constant potential for unanticipated patient safety incidents (PSIs; International Society for Quality in Health Care [1]; World Health Organization [2]). According to the World Health Organization’s International Classification for Patient Safety (ICPS, Version 1.1), PSIs encompass a broad spectrum of events, including those requiring formal reporting, near misses, harmless events, harmful events, and adverse events [3]. The Organization for Economic Cooperation and Development (OECD) estimates that approximately one in 10 hospitalized patients experiences a medical error, with over half of these incidents being preventable [4]. The consequences of PSIs can be severe, resulting in physical harm, psychological distress for patients and families, reputational damage to institutions, and diminished public trust in healthcare [5].

Following a PSI, healthcare professionals are expected to engage in transparent disclosure to patients and their families, including an explanation of the circumstances involved. This process is commonly referred to as “open disclosure.” During this process, healthcare professionals provide clear and accurate explanations, express empathy, and offer reassurance that steps will be taken to ensure the incident does not recur [6]. Open disclosure in PSIs can help patients and their families trust their healthcare professionals and provide them with a positive path toward recovery [7]. This also constitutes an important ethical consideration for healthcare professionals [5]. Furthermore, healthcare institutions can reduce legal dispute costs and the occurrence of similar PSIs, rendering open disclosure essential for improving patients’ quality of life and providing high‐quality healthcare services [8]. However, healthcare professionals generally remain reluctant to engage in open disclosure owing to fear of malpractice lawsuits, reputational damage, and punitive organizational culture [9, 10].

Nurses play a central role in patient safety, accounting for more than half of the hospital workforce and maintaining continuous contact with patients [11]. As first responders to many PSIs, nurses are uniquely positioned to detect, manage, and communicate about safety incidents [12].

Despite this critical role, open disclosure remains a largely voluntary practice, and ethical competence does not invariably translate into actual disclosure behaviors. Ethical competence motivates nurses to recognize disclosure obligations, but organizational climates that generate perceived risks may suppress this motivation, preventing ethical intent from translating into action. Therefore, this study examines whether organizational silence mediates the relationship between nurses’ ethical competence and their perceptions of open disclosure in PSIs.

## 2. Background

Ethical nursing competence involves more than knowledge of professional codes. It requires analyzing ethically complex situations, considering alternative actions, and making norm‐based decisions that prioritize patient rights and safety [13]. Empirical evidence indicates that nurses with higher ethical competence are more likely to support transparent, patient‐centered disclosure following PSIs [14, 15]. Professional guidelines, including the American Nurses Association’s Code of Ethics, necessitate that nurses identify and report unsafe or unethical practices and disclose them to patients [16]. Although prior studies have reported positive associations between ethical competence and open disclosure [9, 15], substantial gaps persist between ethical intent and actual disclosure practice, suggesting the presence of intervening factors.

This study employs organizational silence theory [17] as a framework to understand this discrepancy. The theory conceptualizes silence as a collective phenomenon wherein organizational members deliberately withhold important information due to fear of negative evaluation, retaliation, or other perceived risks shaped by organizational culture and managerial practices. It explains that silence emerges from dynamic interactions between individual beliefs and organizational structures, where fear of negative consequences suppresses voice even among ethically competent professionals. Previous research has identified organizational silence as a key barrier to patient safety communication and error reporting [10, 18]. Nurses’ silence behaviors have been associated with organizational and workplace factors [19, 20], while greater ethical competence appears to correspond with lower levels of silence [14].

Despite growing recognition of both ethical competence and organizational silence as factors influencing patient safety communication, the mechanisms linking these constructs to open disclosure remain underexplored. This study addresses this gap by examining whether organizational silence mediates the relationship between nurses’ ethical competence and their perceptions of open disclosure in PSIs. Based on organizational silence theory [17] and the empirical evidence reviewed above, this study aims to (1) examine the relationships among nurses’ ethical competence, organizational silence, and perceptions of open disclosure and (2) test organizational silence’s mediating role in this relationship. This study provides three significant contributions to nursing management, both in research and in practice. First, it examines organizational silence’s mediating role in the relationship between nurses’ ethical competence and perceptions of open disclosure, addressing a key research gap. Second, it integrates individual‐level ethical capacity and organizational communication climate within a unified framework, responding to calls for multilevel patient safety research. Third, through a detailed examination of these relationships, the study establishes a foundation for developing interventions that simultaneously strengthen ethics education and foster a supportive organizational culture to enhance disclosure practices.

## 3. Methods

### 3.1. Study Design

This study employed a cross‐sectional, theory‐driven correlational design to test a mediation model examining the relationships between nurses’ ethical competence, organizational silence, and perceptions of open disclosure in PSIs.

### 3.2. Participants

Convenience sampling was employed through an online survey administered to registered nurses in Korea. The inclusion criteria were as follows: (1) registered nurses employed in general or tertiary hospitals in South Korea; (2) staff nurses providing direct patient care; and (3) a minimum of 6 months of clinical experience. Recruiting research participants for sensitive topics such as PSIs, ethical competence, and organizational silence presented practical and ethical challenges, so convenience sampling was used. Moreover, recruiting clinical nurses required authorization from healthcare institutions and their active participation during working hours, which made probability sampling difficult to implement. Convenience sampling is frequently used in nursing management research to examine organizational and behavioral phenomena in real‐world clinical settings.

The required sample size was calculated using G∗Power 3.1.9.7 for a multiple regression analysis with *f* ^2^= 0.10, *α* = 0.05, power = 0.95, and 14 predictors, yielding a minimum of 285 participants. After allowing for 10% attrition, the target sample size was 317.

### 3.3. Data Collection and Ethical Considerations

Data were collected between October 14 and November 8, 2024, via an online nursing community. Nurses who expressed interest were provided with the recruitment notice, consent form, and survey link. Of the 317 responses, 14 were excluded for failing to meet the inclusion criteria or showing invalid response patterns (e.g., straight‐lining responses). The final analytic sample included 303 participants (response rate: 96.2%).

The questionnaire included items regarding demographic characteristics (3 items), job‐related factors (3 items), items on PSI‐related factors (5 items), ethical nursing education (2 items), and the main three variables. Permission to use the measurement tools was obtained from the original developers and the Korean translation authors. Ethical approval was obtained from the Institutional Review Board of the university to which the authors of this study belong (No. blinded for review). Participation was voluntary, anonymous, and based on informed electronic consent.

### 3.4. Measurements

Three self‐report instruments were used in this study: (1) ethical nursing competence; (2) organizational silence; and (3) perceptions of open disclosure in PSIs.

#### 3.4.1. Ethical Nursing Competence

Ethical nursing competence was measured using the Ethical Nursing Competence Self‐rating Scale for Clinical Nurses (ENC‐S‐CN), which was developed by Kang and Oh [21] based on the ethical competence framework proposed by Lechasseur et al.’s [13] Ethical Competence Model for Clinical Nurses. A sample item is “I can provide nursing care in an equal relationship with patients.” The ENC‐S‐CN is a 20‐item scale consisting of 5 subscales: ethical behavior (6 items), ethical decision‐making and action (6 items), ethical sensitivity (4 items), ethical reflection (2 items), and ethical knowledge (2 items). Items are rated on a 4‐point Likert scale ranging from 1 (*strongly disagree*) to 4 (*strongly agree*), with higher scores indicating a higher level of ethical nursing competence. Permission to use the measurement tool was obtained from the original developers [21]. Kang and Oh [21] reported a Cronbach’s *α* of 0.89 for the overall scale, with Cronbach’s *α* for the subscales ranging from 0.70 to 0.85. In the present study, Cronbach’s *α* for the overall scale was 0.89.

#### 3.4.2. Organizational Silence

Organizational silence was measured using the Organizational Silence Measurement Scale developed by Dyne et al. [22] and validated for reliability by Kang and Go [23] for use with the Korea Coast Guard. A sample item is “I don’t want to get too involved with the organization, so I don’t propose ideas that could change it.” The scale consists of 13 items divided into three subscales: acquiescent silence (5 items), defensive silence (4 items), and prosocial silence (4 items). Each item is rated on a 5‐point Likert scale ranging from 1 (*not at all*) to 5 (*strongly agree*), with higher scores indicating higher levels of organizational silence within the respective subscale. Permission to use the measurement tools was obtained from the original developers and the Korean translation authors [22, 23]. Dyne et al. [22] did not report reliability coefficients for the scale during its initial development. Kang and Go [23] did not report an overall Cronbach’s *α* but reported this information for the subscales, with scores ranging from 0.85 to 0.92. In this study, the overall Cronbach’s *α* was 0.82.

#### 3.4.3. Perception of Open Disclosure in PSIs

The perception of open disclosure in PSIs was measured using a scale developed by Kim and Lee [9] and adapted from Wagner et al. [24] and Lee et al. [25]. A sample item is “In cases where a medical error has caused serious harm, medical personnel must inform the patient and their guardian of this fact.” The scale consists of 30 items across 6 subscales: open disclosure across harm levels (3 items), open disclosure across situations (6 items), justification for open disclosure (4 items), negative consequences of open disclosure (5 items), positive consequences of open disclosure (6 items), and facilitators of open disclosure (6 items). Items are rated on a 4‐point Likert scale ranging from 1 (*strongly disagree*) to 4 (*strongly agree*). Reverse scoring was applied for negatively worded items, with higher average scores indicating a higher level of perception of open disclosure in PSIs. Permission to use the measurement tool was obtained from the original developers [9]. Kim and Lee [9] reported a Cronbach’s *α* of 0.90 for the total scale at the time of development but did not report subscale reliabilities. In this study, Cronbach’s *α* for the total scale was 0.90.

### 3.5. Statistical Analysis

SPSS 28.0 was used for analysis. Descriptive statistics were used to summarize general characteristics and variable levels. Differences in perceptions by general characteristics were tested using *t*‐tests and a one‐way ANOVA (Scheffé post hoc). Pearson’s correlation coefficient was used to examine the associations among the main variables. To assess potential common method variance, Harman’s single‐factor test was conducted using unrotated principal component analysis. The results showed that multiple factors with eigenvalues greater than 1 were extracted, and the first factor accounted for 23.68% of the total variance, indicating that common method variance was not a serious concern in this study. Age and clinical experience were included as control variables based on prior studies indicating that nurses’ age and clinical experience are associated with patient safety competence and speaking‐up behaviors related to patient safety [26, 27]. Controlling for these variables allowed for a more accurate estimation of the relationships among ethical nursing competence, organizational silence, and perceptions of open disclosure in PSIs. Mediation analysis was performed using the PROCESS Macro Model 4 [28] with 5000 bootstrap resamples. Mediation was considered significant if the 95% CI excluded zero. Confirmatory factor analysis (CFA) was conducted to evaluate the construct validity of the measurement instruments using the SEM module in jamovi [29, 30]. Model fit was assessed using multiple fit indices, including the chi‐square statistic, comparative fit index (CFI), Tucker–Lewis index (TLI), root mean square error of approximation (RMSEA), and standardized root mean square residual (SRMR).

## 4. Results

### 4.1. Measurement Model Validity

CFA was conducted to evaluate the construct validity of the measurement instruments. The measurement model demonstrated acceptable fit to the data (*χ* ^2^ = 4729, df = 1887, *p* < 0.001; CFI = 0.91; TLI = 0.91; RMSEA = 0.071; SRMR = 0.10), supporting the adequacy of the measurement model.

### 4.2. Participant Characteristics and Measured Variables

The results of the frequency analysis and the levels of each variable—including the participants’ demographic and job‐related characteristics, characteristics related to PSIs, and experience with nursing ethics education—are presented in Table 1. An analysis of the demographic characteristics revealed that participants had a mean age of 30.64 years (SD = 4.81) and that the majority were female (94.4%, *n* = 286). Most participants held a bachelor’s degree (81.2%, *n* = 246). The average clinical experience was 6.63 years (SD = 4.68). More than half were employed at general hospitals (54.8%, *n* = 166), and 60.1% (*n* = 182) worked in ward units. Regarding PSI‐related characteristics, 74.9% (*n* = 227) of participants reported experiencing a PSI, with near misses being the most common type (52.9%, *n* = 182). Furthermore, 48.8% (*n* = 148) of participants had received education on open disclosure in PSIs, and of these, 71.6% (*n* = 106) had received the education once within the past year. A total of 152 participants (50.2%) had experience with open disclosure of PSIs. Excluding training on research ethics unrelated to clinical practice, 62.0% (*n* = 188) had received training on nursing ethics. Among this group, 69.7% (*n* = 131) had received such education within the past year just once. The mean scores of variables were as follows: ethical nursing competence, 3.25 ± 0.34 (out of four points); organizational silence, 2.75 ± 0.59 (out of five points); and perception of open disclosure in PSIs, 3.03 ± 0.36 (out of four points).

**TABLE 1:** Participants’ characteristics and measured variables (*n* = 303).

Characteristics	Categories	n	(%)	M ± SD	Range
Age (years)				30.64 ± 4.81	23–52

Sex	Male	17	(5.6)		
	Female	286	(94.4)		

Educational level	3‐year college	21	(6.9)		
	Bachelor’s degree	246	(81.2)		
	≥ Master’s degree	36	(11.9)		

Total working years				6.63 ± 4.68	0.50–31.42

Type of healthcare institution	Tertiary hospital	137	(45.2)		
	General hospital	166	(54.8)		

Current work unit	Ward	182	(60.1)		
	Special units^†^	69	(22.8)		
	Other^‡^	52	(17.2)		

Experience of PSIs	No	76	(25.1)		
	Yes	227	(74.9)		

Types of experienced PSIs^∗^	Near miss	182	(52.9)		
	No harm incident	101	(29.4)		
	Harmful incident (adverse event)	61	(17.7)		

Training in open disclosure in PSIs	No	155	(51.2)		
	Yes	148	(48.8)		

Number of times received training on open disclosure in PSIs (within the past 1 year) (*n* = 148)	0	9	(6.1)	1.22 ± 0.71	0–5
	1	106	(71.6)		
	≥ 2	33	(22.3)		

Experience of open disclosure in PSIs	No	151	(49.8)		
	Yes	152	(50.2)		

Nursing ethics education	No	115	(38.0)		
	Yes	188	(62.0)		

Number of times received education on nursing ethics (within the past 1 year) (*n* = 188)	0	25	(13.3)	1.07 ± 0.67	0–5
	1	131	(69.7)		
	≥ 2	32	(17.0)		

Ethical nursing competence				3.25 ± 0.34	1–4

Organizational silence				2.75 ± 0.59	1–5

Perception of open disclosure in PSIs				3.03 ± 0.36	1–4

Abbreviations: M = mean; SD = standard deviation.

^∗^Multiple responses were permitted.

^†^Special units: intensive care unit, emergency room, operation room/anesthesia/recovery room.

^‡^Others: outpatient department, examination room, delivery/newborn unit, physician assistant.

### 4.3. Comparison of Perception of Open Disclosure in PSIs According to General Characteristics

The differences in perceptions of open disclosure in PSIs according to participants’ general characteristics are summarized in Table 2. Significant differences were observed in the type of healthcare institution (*t* = −3.07, *p* = 0.002), current working unit (*F* = 3.93, *p* = 0.021), PSI experience (*t* = −3.53, *p* = 0.001), training experience in open disclosure in PSIs (*t* = 2.92, *p* = 0.004), number of such training sessions in the past year (*F* = 3.35, *p* = 0.038), and nursing ethics education experience (*t* = 2.70, *p* = 0.007). Perception scores were higher among nurses in general hospitals than those in tertiary hospitals, among those without PSI experience, and among those who had received training in open disclosure or nursing ethics education. Participants who had attended at least one training session on open disclosure in the past year scored higher than those who did not. No significant differences were found in the perception scores based on the number of nursing ethics education sessions completed in the past year.

**TABLE 2:** Comparison of perception of open disclosure in PSIs according to general characteristics (*N* = 303).

Characteristics	Categories	M±SD	t/F	*p*	Scheffé́
Sex	Male	2.91 ± 0.32	−1.42	0.158	
	Female	3.03 ± 0.36			

Educational level	3‐year college	2.91 ± 0.30	1.67	0.190	
	Bachelor’s degree	3.04 ± 0.35			
	≥ Master’s degree	2.98 ± 0.38			

Type of healthcare institution	Tertiary hospital	2.96 ± 0.30	−3.07	0.002	
	General hospital	3.08 ± 0.38			

Current working unit	Ward^a^	3.07 ± 0.37	3.93	0.021	n/a
	Special units^†^ ^b^	2.96 ± 0.34			
	Other^‡^ ^c^	2.95 ± 0.29			

Experience of PSIs	No	3.16 ± 0.40	−3.53	0.001	
	Yes	2.98 ± 0.33			

Training on open disclosure in PSIs	No	2.97 ± 0.33	2.92	0.004	
	Yes	3.09 ± 0.37			

Number of times received training on open disclosure in PSIs (within the past 1 year) (*n* = 148)	0^a^	2.83 ± 0.23	3.35	0.038	*a* < *b*
	1^b^	3.13 ± 0.38			
	≥ 2^c^	3.03 ± 0.32			

Experience of open disclosure in PSIs	No	3.02 ± 0.38	0.51	0.608	
	Yes	3.04 ± 0.33			

Nursing ethics education	No	2.96 ± 0.33	2.70	0.007	
	Yes	3.07 ± 0.36			

Number of times received education on nursing ethics (within the past 1 year) (*n* = 188)	0	2.91 ± 0.34	3.03	0.051	
	1	3.09 ± 0.36			
	≥ 2	3.13 ± 0.37			

Abbreviations: M = mean; SD = standard deviation.

^†^Special units: intensive care unit, emergency room, operation room/anesthesia/recovery room.

^‡^Others: outpatient department, examination room, delivery/newborn unit, physician assistant.

^a, b,^ and ^c^ are used to denote the results of Scheffé’s post hoc test (a < b).

### 4.4. Correlation Among Main Variables

The results of the correlation analysis, which examined the relationships among participants’ general characteristics (age and total clinical experience), ethical nursing competence, organizational silence, and perceptions of open disclosure in PSIs, are presented in Table 3.

**TABLE 3:** Correlations among age, total working years, ethical nursing competence, organizational silence, and perceptions of open disclosure in PSIs (*N* = 303).

**Variables**	**Age**	**Total working years**	**Ethical nursing competence**	**Organizational silence**	**Perception of open disclosure in PSIs**
			***r*(** **p** **)**		

Age	1				
Total working years	0.85 (< 0.001)	1			
Ethical nursing competence	−0.19 (0.001)	−0.24 (< 0.001)	1		
Organizational silence	< 0.01 (0.960)	−0.03 (0.597)	−0.28 (< 0.001)	1	
Perception of open disclosure in PSIs	−0.12 (0.035)	−0.20 (0.039)	0.60 (< 0.001)	−0.36 (< 0.001)	1

Perceptions of open disclosure in PSIs were significantly negatively correlated with age (*r* = −0.12, *p* = 0.035), total clinical experience (*r* = −0.20, *p* = 0.039), and organizational silence (*r* = −0.36, *p* < 0.001) and positively correlated with ethical nursing competence (*r* = 0.60, *p* < 0.001). Ethical nursing competence was significantly negatively correlated with age (*r* = −0.19, *p* = 0.001), total working years (*r* = −0.24, *p* < 0.001), and organizational silence (*r* = −0.28, *p* < 0.001) and significantly positively correlated with perceptions of open disclosure in PSIs (*r* = 0.60, *p* < 0.001). Organizational silence was significantly negatively correlated with ethical nursing competence (*r* = −0.28, *p* < 0.001) and perceptions of open disclosure in PSIs (*r* = −0.36, *p* < 0.001).

### 4.5. Mediating Role of Organizational Silence on the Relationship Between Ethical Nursing Competence and Perception of Open Disclosure in PSIs

All regression assumptions (linearity, normality, homoscedasticity, independence, and absence of multicollinearity) were met. To enhance the accuracy of the statistical analysis, variables that indicated significant differences in the perception of open disclosure in PSIs in the preliminary analysis, such as type of healthcare institution, current working unit, experience with PSIs, experience of ethical nursing education, and training on open disclosure in PSIs, were controlled for as covariates. However, the number of times they had received training on open disclosure in PSIs was excluded due to the limited number of participants with such experience. Considering the strong correlation between age and total clinical experience, only total clinical experience was included as a covariate to avoid multicollinearity.

**FIGURE 1:**
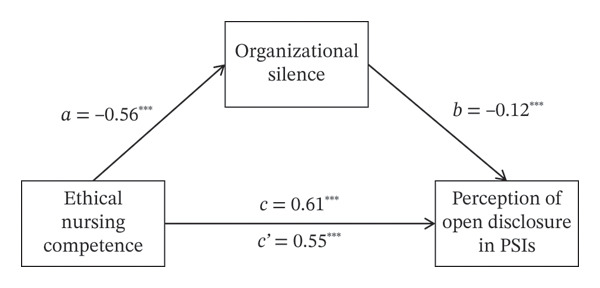
Mediation model of organizational silence linking ethical nursing competence and perception of open disclosure in PSIs among nurses. ^∗∗∗^ *p* < 0.001.

First, an analysis of the effect of ethical nursing competence on organizational silence revealed that ethical nursing competence significantly reduced organizational silence (*B* = −0.56, *p* < 0.001). Next, with the perception of open disclosure in PSIs as the dependent variable and ethical nursing competence and organizational silence as predictor variables, ethical nursing competence was found to have a positive effect on the perception level (*B* = 0.55, *p* < 0.001), while organizational silence had a negative effect (*B* = −0.12, *p* < 0.001). Investigating the mediating effect revealed that the indirect effect of ethical nursing competence on the perception of open disclosure in PSIs was *B* = 0.07 (95% CI [0.029, 0.111]), which was significant as the 95% confidence interval did not include “0.” Therefore, organizational silence was confirmed as a mediating variable in the relationship between ethical nursing competence and the perception of open disclosure in PSIs. That is, a dual pathway existed: Ethical nursing competence directly increased the perception of open disclosure in PSIs while also indirectly enhancing the perception level by reducing organizational silence. The total effect was confirmed as *B* = 0.61 (95% CI [0.506, 0.721]). The model explained 42.7% of the variance in perception of open disclosure in PSIs. The mediation model for organizational silence is shown in Figure 1 and Table 4.

**TABLE 4:** The mediating effect of organizational silence in the relationship between ethical nursing competence and perception of open disclosure in PSIs.

	**Independent variable**	**Dependent variable**	**B**	**SE**	**β**	**p**	**95% CI**	
							**Lower**	**Upper**

	Ethical nursing competence	Organizational silence	−0.56	0.11	−0.32	< 0.001	−0.78	−0.34
	Organizational silence	Perception of open disclosure in PSIs	−0.12	0.03	−0.20	< 0.001	−0.18	−0.06
Direct effect (c’)	Ethical nursing competence	Perception of open disclosure in PSIs	0.55	0.06	0.52	< 0.001	0.44	0.66
Indirect effect (ab)	Ethical nursing competence ⟶ Organizational silence ⟶ Perception of open disclosure in PSIs	0.07	0.02			0.03	0.11	
Total effect		0.61	0.06		< 0.001	0.51	0.72	
(*c* = *c*’ + ab)								

*Note:* Covariates = type of healthcare institution, current working unit, experience with PSIs, experience of ethical nursing education, and training on open disclosure in PSIs.

Abbreviation: SE = standardized error.

## 5. Discussion

This study analyzed the mediating role of organizational silence on the relationship between ethical nursing competence and the perception of open disclosure in PSIs. Based on the findings, the main points of discussion are as follows:

The results show that participants without PSI experience had significantly higher perceptions of open disclosure in PSIs than those with PSI experience. This finding suggests that negative experiences encountered when actual PSIs occur act as greater barriers to communication than the perceptions held by those who have not experienced such incidents. Nurses appeared to be highly aware of and concerned about the negative aspects of disclosing and communicating openly about PSIs. Although nurses recognize their professional obligation to communicate with patients or guardians about PSIs as healthcare providers, various factors may hinder open information sharing, including hostile reactions from patients or families, concerns about legal consequences, criticism from colleagues, and the resulting psychological burden [9, 31]. The robustness of these findings is further supported by the acceptable construct validity of the measurement model confirmed through CFA.

Our findings revealed that high ethical nursing competence demonstrated a significant positive correlation with the perceptions of open disclosure in PSIs and a negative correlation with organizational silence. These findings are consistent with and supported by several previous studies that explored similar relationships in healthcare settings [14, 15, 18]. Nevertheless, an interesting and somewhat counterintuitive finding emerged: We discovered that longer working experience was associated with lower perceptions of open disclosure in PSIs, suggesting that years of practice may actually diminish nurses’ willingness to communicate openly about safety incidents. This phenomenon may be closely related to complex administrative and organizational factors that have been identified and discussed in previous research [32, 33]. As work experience accumulates, individuals inevitably develop and maintain diverse social relationships within their workplace, creating intricate networks of professional connections and dependencies. Consequently, the tendency to prefer prosocial silence and avoid conflict—considering organizational interests and reputation—may explain why honestly disclosing PSIs and apologizing has become less common [34].

The main finding of this study is that organizational silence has a significant mediating effect on the relationship between ethical nursing competence and the perceptions of open disclosure in PSIs. This crucial discovery demonstrates the pivotal and multifaceted role of organizational silence by confirming not only its direct effect on nurses’ perceptions of open disclosure in PSIs but also its indirect mediating effect that helps explain the pathway through which ethical competence ultimately impacts disclosure behaviors. This mediating relationship provides valuable insight into the mechanisms underlying nurses’ communication patterns regarding PSIs. Previous research has shown that nurses’ ethical integrity influences organizational silence [35, 36], and that organizational silence affects the open disclosure or reporting of PSIs [37, 38]. Other studies have also found associations between ethics and the communication or reporting of PSIs [15, 39].

From a theoretical perspective, this study makes an important contribution by extending organizational silence theory [17] to the patient safety context. While the original theory primarily focused on upward communication failures in organizational settings, these findings demonstrate that organizational silence operates as a mediating mechanism in the translation of individual ethical competence into patient‐centered disclosure behaviors. This extends understanding beyond viewing ethical competence and organizational climate as parallel predictors, revealing that organizational silence functions as an intervening process through which ethical competence is either facilitated or suppressed. By integrating individual‐level ethical competence with organizational‐level communication climate, this study offers a more nuanced theoretical framework for understanding how professional ethics operate within organizational contexts. These findings suggest that organizational silence theory provides a valuable lens for explaining not only why employees withhold information from management, but also how organizational climates influence the enactment of professional ethical obligations in patient care.

Therefore, to enhance the perceptions of open disclosure in PSIs, future research is needed to develop and educate nurses on systematic ethical reflection programs drawing on prior studies that confirm the effectiveness of reflection for improving ethical nursing competence [40, 41]. Additionally, strategies for improving organizational culture must be explored to foster an environment in which nurses can freely express their opinions and suggest improvements. Nurses’ tendency to engage in prosocial silence [19, 42] may help them maintain relationships with other hospital staff but can also conceal organizational issues. It can also negatively impact patient health, social values, and nursing ethics. Therefore, a nonpunitive and open organizational atmosphere must be created [43], which requires multidimensional support from nursing organizations and medical institutions.

The limitations of this study were as follows: First, it did not examine the differences in perceptions of open disclosure in PSIs according to incident type, which restricted the interpretation of the results. Future studies should consider incident characteristics to provide more nuanced findings. Second, owing to the limited number of previous studies addressing the same variables, the interpretation of causal relationships remains constrained. Replication studies with diverse research designs are needed to strengthen the evidence base. Third, the cross‐sectional design of this study made it difficult to capture changes in ethical nursing competence, organizational silence, and perceptions of open disclosure over time. Therefore, further longitudinal studies are recommended. Fourth, while this study focused on organizational silence as the primary mediating mechanism, other potential mediators such as moral courage, psychological safety, or fear of retaliation were not examined. Future research should explore multiple mediating pathways to provide a more comprehensive understanding of how ethical competence translates into disclosure behaviors.

Despite these limitations, this study empirically identified the mediating role of organizational silence in the relationship between ethical nursing competence and perceptions of open disclosure in PSIs. This highlights the importance of organizational silence not only as a direct factor but also as an indirect pathway influencing open disclosure. The findings suggest that both individual‐level ethical competence and organizational‐level factors should be considered when seeking to improve perceptions of open disclosure. These results provide a basis for further research on strategies to strengthen ethical competence, reduce organizational silence, and foster an open culture in clinical practice.

## 6. Conclusion

This study demonstrated that organizational silence mediates the relationship between ethical nursing competence and nurses’ perceptions of open disclosure in PSIs. This finding extends organizational silence theory by demonstrating how organizational climates shape the translation of individual ethical capacity into patient‐centered communication behaviors. Improving patient safety requires a dual focus: strengthening nurses’ ethical competence through systematic training and fostering organizational environments that minimize silence and support open dialogue.

## 7. Implications for Nursing Management

The findings suggest that nursing management should prioritize developing nurses’ ethical competence through targeted education and training programs, as higher ethical competence directly contributes to reduced organizational silence and increased support for the open disclosure of PSIs. Managers must actively create psychologically safe environments that encourage open communication by implementing nonpunitive reporting systems and establishing clear protocols for incident disclosure. Leadership development is essential to equip managers with the skills to recognize and address organizational silence while fostering a transparent communication culture. Additionally, integrating measures of ethical competence and open communication behaviors into performance evaluation and recognition programs can reinforce organizational commitment to patient safety transparency. Regular monitoring of organizational climate regarding these factors will enable continuous improvement of management strategies to support both ethical practices and patient safety outcomes.

## Funding

No funding was received for this research.

## Disclosure

This study is based on the first author’s master’s thesis.

## Conflicts of Interest

The authors declare no conflicts of interest.

## Data Availability

The data that support the findings of this study are available on request from the corresponding author. The data are not publicly available due to privacy or ethical restrictions.
